# Exogenous Glycinebetaine Promotes Soil Cadmium Uptake by Edible Amaranth Grown during Subtropical Hot Season

**DOI:** 10.3390/ijerph15091794

**Published:** 2018-08-21

**Authors:** Wei-Qing Yao, Yong-Kang Lei, Ping Yang, Qu-Sheng Li, Li-Li Wang, Bao-Yan He, Zhi-Min Xu, Chu Zhou, Han-Jie Ye

**Affiliations:** 1 Guangdong Polytechnic of Environmental Protection Engineering, Foshan 528216, China; yaofast1@163.com; 2 School of Environment, Key Laboratory of Environmental Pollution and Health of Guangdong Province, Jinan University, Guangzhou 511443, China; leiyongkang0611@163.com (Y.-K.L.); yangp35@jnu.edu.cn (P.Y.); wanglili@jnu.edu.cn (L.-L.W.); thbyan@jnu.edu.cn (B.-Y.H.); cloris59420@163.com (Z.-M.X.); zhouchuzhc@163.com (C.Z.); a3192396@163.com (H.-J.Y.)

**Keywords:** lycine, high temperature, Cd, *Amaranthus*, chlorophyll, pectin

## Abstract

Exogenous glycinebetaine treatment is an effective measure for preventing crops from being exposed to drought and high temperature; however, the effects of this approach on the soil Cd uptake and accumulation by crops remain unclear. Pot experiments were conducted in this study to analyze the effect of glycinebetaine on the soil Cd uptake and accumulation by edible amaranth cultivated in Cd-contaminated soil. Results revealed that after exogenous glycinebetaine treatment on amaranth leaves during the vigorous growth period, the plant biomass, the Cd concentrations in the roots and shoots, and the Cd translocation factor (TF) were significantly higher than those of the control group. The highest Cd concentrations in the roots and shoots and the TF were higher by 91%, 96% and 23.8%, respectively, than the corresponding values in the control group. In addition, exogenous glycinebetaine treatment significantly increased leaf chlorophyll content and promoted the photosynthesis of edible amaranth. Consequently, the contents of soluble sugar, dissolved organic carbon, and low-molecular-weight organic acids significantly increased in the rhizosphere, resulting in Cd mobilization. Significant positive correlations were observed among the contents of leaf chlorophyll, Mg, Fe, pectin and Ca. Given that Cd shares absorption and translocation channels with these elements, we speculated that the increased leaf chlorophyll and pectin contents promoted the absorption and accumulation of Mg, Fe and Ca, which further promoted the absorption and translocation of Cd. These results indicated that exogenous glycinebetaine treatment during hot season would aggravate the health risks of crops grown in Cd-contaminated soils.

## 1. Introduction

Glycinebetaine (GB) is one of the main organic osmoregulators of plants. Plants can be exposed to various stresses, such as salt, drought, or high temperature, and glycinebetaine can exert osmoregulation synergistically with proline and soluble sugars to enhance plant resistance [1,2]. Glycinebetaine enhances the resistance of plants to salt [3,4,5], drought [6,7], and cold stress [8].

Exogenous glycinebetaine can also effectively mitigate high-temperature stress in crops [9,10,11]. Previous studies found that exogenous glycinebetaine alleviates the decline in photosynthetic rate due to stomatal limitation and attenuates the effect of high temperature on the photosynthetic physiological processes of plants [12,13]. Kanechi et al. [9] reported that glycinebetaine could significantly increase the chlorophyll content in tomato plants under high-temperature stress and enhance photosynthesis. Glycinebetaine could improve the high-temperature resistance of transgenic wheat by protecting the repair cycle of photosystem II [14]. In addition, exogenous glycinebetaine treatment promotes high-temperature resistance and growth in transgenic *codA* genes (a gene encodes choline oxidase, the enzyme that converts choline to glycinebetaine) of *Arabidopsis* [15] and transgenic BADH genes (a gene encoding betaine aldehyde dehydrogenase) of wheat [16]. Therefore, exogenous glycinebetaine is introduced in some regions to resist high temperatures and promote crop growth. In recent years, many soils have been contaminated by heavy metals, such as Cd, due to human activities (such as smelt, long-term application of chemical fertilizer, sewage irrigation). Cd can easily enter the food chain through crop enrichment, posing a health risk to humans [17,18]. Although glycinebetaine treatment can protect crops against drought and high-temperature stress, its effects on the soil Cd uptake and accumulation by crops remain unclear. Previous studies reported that exogenous glycinebetaine treatment could significantly inhibit the Cd uptake by tobacco cells [19] and increase the Cd concentration in maize seeds [20]. Exogenous glycinebetaine treatment could significantly alleviate the Cd stress of mung bean seedlings [21], rice seedlings [22], and cotton seedlings [23] by enhancing the activity of antioxidant enzymes. However, these studies failed to clarify the mechanism by which glycinebetaine affects the soil Cd uptake and accumulation by crops.

The Cd concentration in agricultural soils in the Pearl River Delta area far exceeds the standard of “Farmland environmental quality evaluation standards for edible agricultural products (HJ 332-2006, China)” (Cd ≤ 0.3 mg kg^−1^, in soil with pH ≤ 7.5), such that planting crops in this subtropical region presents environmental risks [24,25]. Moreover, the increased transpiration during the hot season relative to that during cool season would intensify the soil Cd uptake and accumulation by crops, thereby aggravating the environmental risks [26]. Therefore, this paper assumed that exogenous glycinebetaine treatment on leaves may affect the soil Cd uptake and accumulation by vegetables in the subtropical hot season. To verify this hypothesis, experiments were conducted using edible amaranth, which is widely grown in the subtropical hot season and has a high edible value. The specific objectives were as follows: (1) to determine the Cd concentrations in the rhizosphere soil solution, the roots, and the shoots after exogenous glycinebetaine treatment of different concentrations on the leaves at different growth stages of edible amaranth; (2) to analyze the changes in the soluble sugar, dissolved organic carbon (DOC), and low-molecular-weight organic acids (LMWOAs) in the rhizosphere soil solution of amaranth after exogenous glycinebetaine treatment, as well as their relationship to the Cd mobilization in the rhizosphere soil; and (3) to investigate the changes in pectin, chlorophyll, Ca, Mg, and Fe contents, as well as their relationship to the Cd uptake and accumulation by edible amaranth after exogenous glycinebetaine treatment.

## 2. Materials and Methods

### 2.1. Experimental Crops and Soil

Liuye edible amaranth (*Amaranthus mangostanus* L.) was selected as the study object, and the seeds were purchased in the local market of Guangzhou, China. Glycinebetaine anhydrous was acquired from Sigma. The potting soil for the experiment was collected from the vegetable garden soil in the suburbs of Guangzhou City, Guangdong Province. The soil was irrigated with sewage 20 years ago. The experimental soil had a pH of 6.35, a total organic carbon of 35.4 g kg^−1^, a cation exchange capacity of 20.86 cmol kg^−1^, and a Cd concentration of 1.81 ± 0.10 mg kg^−1^, which was significantly higher than the standard Cd concentration of 0.3 mg kg^−1^ in the “Farmland environmental quality evaluation standards for edible agricultural products” (HJ 332-2006). Therefore, the experimental soil was a Cd-contaminated soil. The soils of the tillage layer (0–15 cm) were collected and transported to a glasshouse for natural drying. After the moisture content was determined, the soil was ground and sieved (2 mm) and thoroughly mixed for the pot experiments.

The pot experiments were performed in a glasshouse located on the campus of Jinan University (120° 58′ E, 23° 58′ N). Accurately weighed 2.20 kg soil samples were placed in plastic pots (pot height: 15 cm; pot base diameter: 15 cm). The experimental amaranth seeds were soaked in disinfectant solution (0.05% carbendazim) for 25 min and then rinsed with tap water and deionized water. Then, the seeds were sown in nylon mesh in the plastic pots. Plastic trays were placed under the pots. The water content was maintained at about 75% of the field moisture capacity, and the percolation water produced by irrigation was recharged to the soil. During the experiment, the temperature in the greenhouse during daytime was maintained at 36–42 °C for 6 h (from 10 a.m. to 4 p.m.).

The experimental design consisted of two series. For Series 1, potted amaranth leaves were subjected to exogenous glycinebetaine treatment at five levels (1, 5, 10, 20 and 50 mmol L^−1^) at different growth stages (early growth, Day 18; vigorous growth, Day 30). Glycinebetaine was applied on the leaves once every 2 days for three times, and deionized water was used as the blank control (glycinebetaine: 0 mmol L^−1^). Each treatment was repeated five times, and the total number of pots was 60. This series was established to investigate the soil Cd uptake and accumulation by amaranth after foliar exogenous glycinebetaine treatment of different concentrations at different growth stages. For Series 2, according to the results of Series 1, exogenous glycinebetaine treatment was applied on the amaranth leaves at the vigorous growth stage at three levels (5, 10 and 50 mmol L^−1^) and a blank control (0 mmol L^−1^). The other experimental conditions were the same as those of Series 1, and the total number of pots was 20. The purpose of this series was to examine the changes in the rhizosphere soluble sugar, DOC, LMWOAs, and in the leaf chlorophyll and pectin. The amaranth in both experiment series was grown for 50 days and then harvested at 6:00 AM.

### 2.2. Sample Determination

After the plants in Series 1 were washed clean and dried with filter paper, their fresh weight was measured. The plants were placed in an oven and de-enzymed at 105 °C for 15–20 min. Then, the temperature was maintained at 70–80 °C until a constant weight was achieved, and the dry weight was measured. The plants were pulverized with a plant grinder. Accurately weighed 0.3 g powder samples were digested with HNO_3_ in a microwave digestion instrument. The digested solution was adjusted to 25 mL with deionized water and was used to determine the Cd concentrations in the roots and the shoots.

After the plants in Series 2 were washed clean and dried with filter paper, their fresh weight was measured. A selected part of the root, stem, and leaf was quick-frozen in liquid nitrogen and then stored at 0–4 °C to measure the soluble sugar and pectin contents. The soluble sugar content of root was determined using anthrone colorimetry [27], and the total pectin content of leaf was obtained using galacturonic acid colorimetry [28]. The remaining samples were dried, and a part leaf was used to determine the chlorophyll content. Chlorophyll was extracted by using 95% ethanol, and its content was determined by UV mini-1240 UV–Vis spectrophotometer (Shimadzu, Kyoto, Japan) [29]. Parts of the dried samples were ground and digested to determine the concentration of Cd, Ca, Mg, and Fe in the root, stem, and leaf. At the same time as the plants were harvested, the rhizosphere soil in the nylon mesh was placed in sealed bags. The rhizosphere soil solution was collected using the high-speed centrifugation method [30]. The soil solution was filtered twice with a 0.45-μm filter, and then stored in a −80 °C freezer for determination of the contents of Cd, soluble sugar, DOC, and LMWOAs [31].

The Cd concentration was measured by graphite furnace atomic absorption spectrophotometry (GF-AAS-7000A, Shimadzu, Kyoto, Japan), and the Ca, Mg, and Fe contents were determined by inductively coupled plasma optical emission spectrometry (ICP-OES Avio200, PerkinElmer, Waltham, MA, USA). The plant standard sample (GSV-1 national standard reference material) was used for quality control, and the recovery rate was 85–110%. The DOC content was measured using a dissolved organic carbon analyzer (Toc-Vcsh, Shimadzu, Kyoto, Japan). LMWOAs were measured by gradient elution ion chromatography (ICS-1100, DIONEX, Sunnyvale, CA, USA).

### 2.3. Data Analysis

The translocation factor (TF) was calculated using Equation (1):

TF_Cd_ = Cd_shoot_/Cd_root_ (1)

Cd_root_ (fresh weight, µg g^−1^) and Cd_shoot_ (fresh weight, µg g^−1^) represent the Cd concentration in the amaranth roots and shoots, respectively.

The total Cd amount accumulated in plant (Cd_accu_ (fresh weight, µg pot^−1^)) was calculated using Equation (2):

Cd_accu_ = Cd_root_ × W_root_ + Cd_shoot_ × W_shoot_ (2)

W_root_ (g pot^−1^) and W_shoot_ (g pot^−1^) represent the weight of amaranth roots and shoots in each pot, respectively.

The total amount of mobilized Cd in the rhizosphere soil (Cd_total_ (µg pot^−1^)) was calculated using Equation (3):

Cd_total_ = (Cd_accu_ + Cd_rhizo_ × W_rhizo_) (3)

Cd_rhizo *_ (µg g^−1^ dry soil weight) represents the Cd concentration in the amaranth rhizosphere soil solution, and W_rhizo_ (g pot^−1^) represents the dry weight of the rhizosphere soil in each pot.

Statistical analysis was conducted using SPSS 19.0 (IBM, Armonk, NY, USA). The kurtosis and skewness of each sample was in a reasonable range (−1, 1), and a Q-Q plot indicated that each point approximately surrounded a straight line, indicating that each sample was approximately normal distribution. Data were tested at significance levels of *p* < 0.05 by student’s *t*-test and ANOVA Duncan test. Origin 9.0 software (OriginLab, Northampton, MA, USA) was used to draw the graphs.

## 3. Results and Discussion

### 3.1. Biomass and Cd Concentration in Edible Amaranth after Exogenous Glycinebetaine Treatment at Two Growth Stages

**Figure 1:**
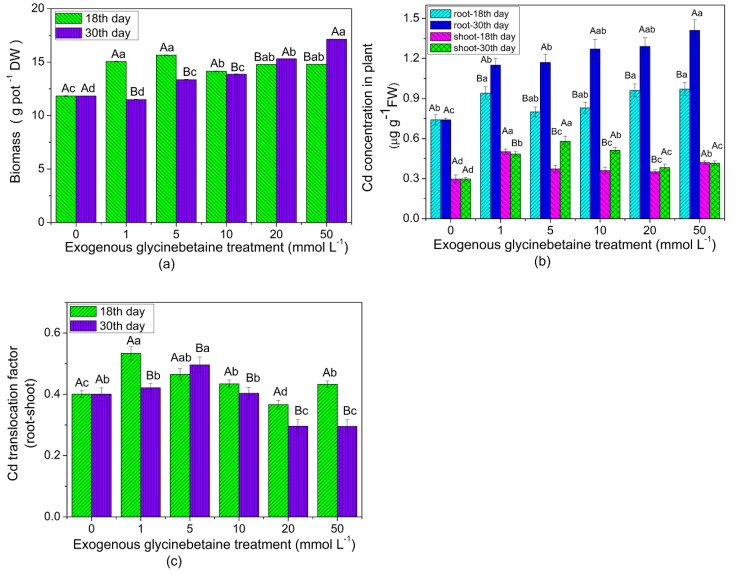
Biomass (**a**), Cd concentration in plant (**b**), and Cd translocation factor (**c**) under exogenous glycinebetaine treatment at two growth stages of edible amaranth.

Figure 1 shows the biomass, Cd concentration in the plant, and the Cd TF after exogenous glycinebetaine treatment on the leaves at two growth stages of amaranth. The results revealed that the biomass of most treated plants was significantly higher than that of the control (Figure 1a), indicating that glycinebetaine can alleviate high-temperature stress and promote plant growth. This finding was consistent with the results of Kanechi et al. [9]. As shown in Figure 1b, when exogenous glycinebetaine treatments of different concentrations were applied to the amaranth leaves of the same growth stage, the Cd concentrations in the roots and the shoots were significantly higher (*p* < 0.05) than those of the control, and the highest Cd concentrations in the roots and the shoots were higher by 91% and 96%, respectively, than that in the control. The shoots of amaranth are the main edible parts. The Cd concentrations in the roots and the shoots of plants in the vigorous growth stage were significantly higher (*p* < 0.05) than those of the early growth stage. After exogenous glycinebetaine treatment on the leaves of plants in the early and vigorous growth stages, the average Cd concentrations in the shoots were 0.40 and 0.47 µg g^−1^, respectively, which were 33–57% higher than those in the control (Cd concentration: 0.3 µg g^−1^). This result implied that the exogenous glycinebetaine treatment on the leaves greatly intensified the human health risk of edible amaranth. The TF of Cd from the roots to the shoots significantly increased under 1 and 5 mM treatments relative to that of the control, with the highest increase recorded at 33.2%. However, the change was insignificant, and the TF even significantly decreased under a high-concentration treatment (Figure 1c). This finding showed that a low-concentration glycinebetaine treatment promoted the translocation of Cd from the roots to the shoots, whereas a high concentration inhibited the translocation. In view of the greater effect of glycinebetaine treatment on the Cd uptake and accumulation at the vigorous growth stages, the following experiments were conducted under glycinebetaine treatment on the leaves of amaranth during the vigorous growth stage.

The capital letters (A and B) indicate significant differences between two growth stages under the same treatment for root or shoot (student’s *t*-test, *p* < 0.05). The lowercase letters (a, b, c and d) indicate significant differences between different treatments in the same growth stages (ANOVA Duncan test, *p* < 0.05). The number of each group sample was five (*n* = 5).

### 3.2. Soluble Sugar Content in the Amaranth Roots, Contents of Soluble Sugar, DOC, and LMWOAs in the Rhizosphere Solution and their Relationship with Cd Mobilization in Rhizosphere Soil

**Figure 2:**
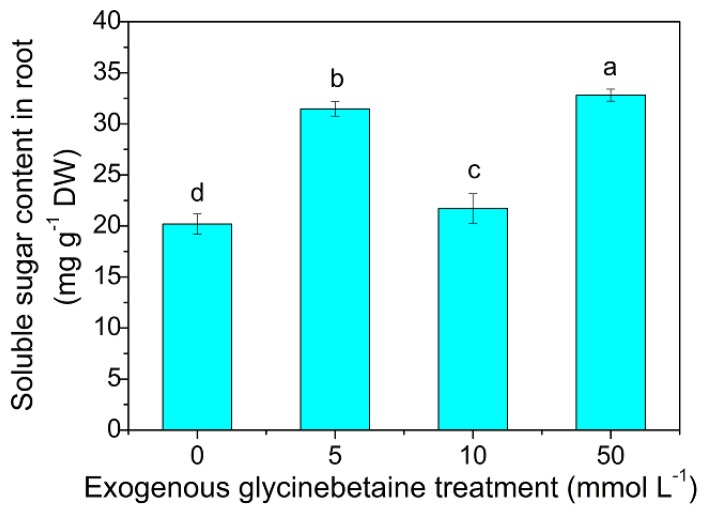
Soluble sugar content in the roots of control and exogenous glycinebetaine treatment plants. Different lowercase letters indicate significant difference between treatments (ANOVA Duncan test, *p* < 0.05). The number of each group sample was five (*n* = 5).

As shown in Figure 2, the soluble sugar content in the roots significantly increased (*p* < 0.05) after exogenous glycinebetaine treatment on the leaves. When the glycinebetaine concentration was 50 mM, the soluble sugar content in the roots was the highest, which was 62.52% higher than that of the control. Significant differences (*p* < 0.05) were observed in the effects of different concentrations of glycinebetaine treatments. Gao et al. [32] reported that exogenous glycinebetaine treatment increases the contents of proline and soluble sugar in tomato plants. High temperatures promote leaf transpiration and cause water loss, thereby closing the stomata and inhibiting photosynthesis. Exogenous glycinebetaine can quickly seep into plant organs, preserve the osmotic potential in the leaves, alleviate the stomatal limitation caused by high-temperature stress, and, therefore, enhance photosynthesis and increase the soluble sugar content in the roots [8].

Table 1 shows the total mobilized Cd amount in the rhizosphere soils and the changes in the soluble sugar, DOC, and LMWOAs contents in the rhizosphere soil solution after exogenous glycinebetaine treatment. After exogenous glycinebetaine treatment, Cd_total_ significantly increased (*p* < 0.05), and the mean value was 1.66 times higher than that of the blank control. Table 1 also shows that glycinebetaine significantly increased the DOC, LMWOAs, and soluble sugar contents (*p* < 0.05) in the rhizosphere soil solution, and the corresponding mean values were 1.37, 3.73 and 2.36 times higher than that of the blank control, respectively. A significant positive correlation existed between Cd_total_ and DOC, LMWOAs, and soluble sugars at the 0.05 level (Table 2). LMWOAs are part of the DOC and not only directly chelate Cd to enhance soil Cd mobility but also acidify the rhizosphere soils to improve the bioavailability of heavy metals [33,34]. Therefore, LMWOAs play a key role in the metal mobilization in the rhizosphere soil. The literature confirmed that glucose metabolism produces more LMWOAs [30]. We speculate that exogenous glycinebetaine treatment promotes the photosynthesis of amaranth and increases the soluble sugar content of root, which further increases the total contents of DOC, LMWOAs, and soluble sugar in the rhizosphere soil. In addition, DOC, LMWOAs, and soluble sugars provide nutrients to the rhizosphere microorganisms, increasing their abundance. In turn, the rhizosphere microorganisms enhance the bioavailability of Cd by dissolving insoluble Cd through their metabolism.

**Figure 3:**
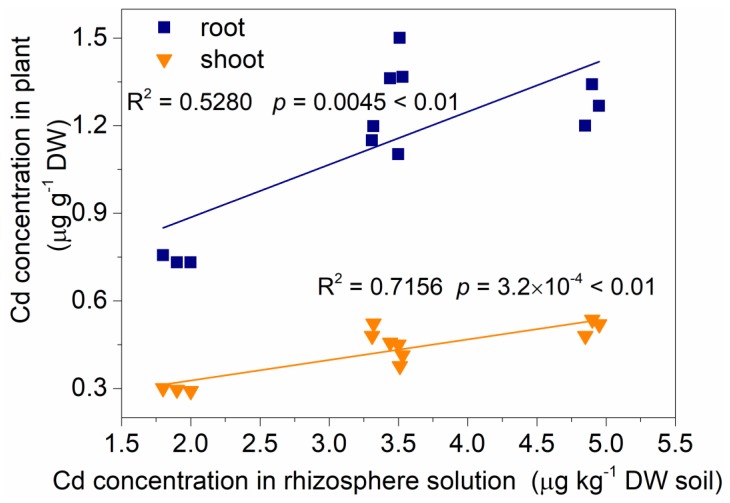
Correlation of Cd_rhizo_ with Cd_root_, and Cd_shoot_ in amaranth after exogenous glycinebetaine treatment.

As shown in Figure 3, after exogenous glycinebetaine treatment, Cd_rhizo_ presented a significant positive correlation with Cd_root_ and Cd_shoot_ in amaranth (R^2^ = 0.5280, 0.7156; *p* < 0.01, 0.01), indicating that Cd mobilization in the rhizosphere soil played an important role in the Cd accumulation in the roots and the shoots of edible amaranth. This finding was consistent with the result of Xu et al. [35].

### 3.3. Changes in Ca, Mg and Fe Contents in the Leaves and their Relationship with Cd Uptake by Edible Amaranth

**Figure 4:**
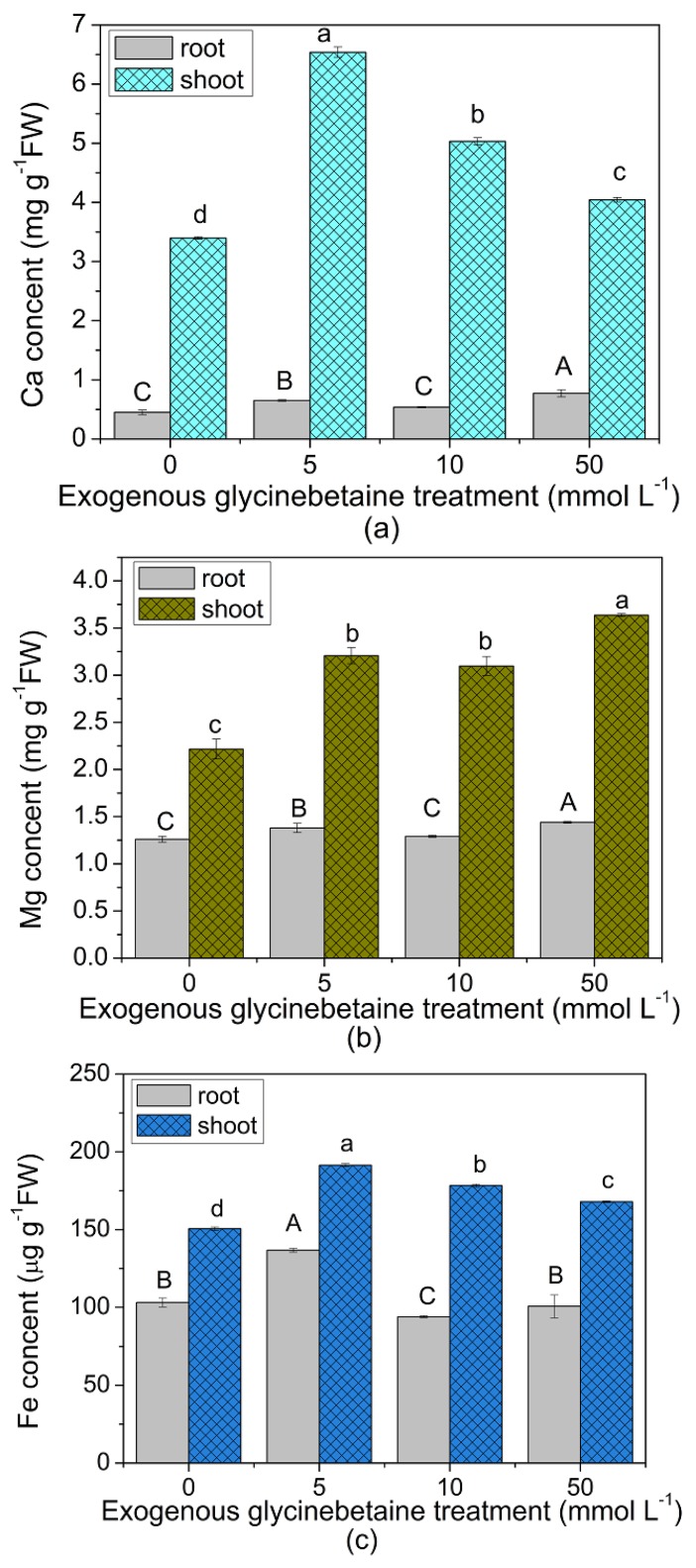

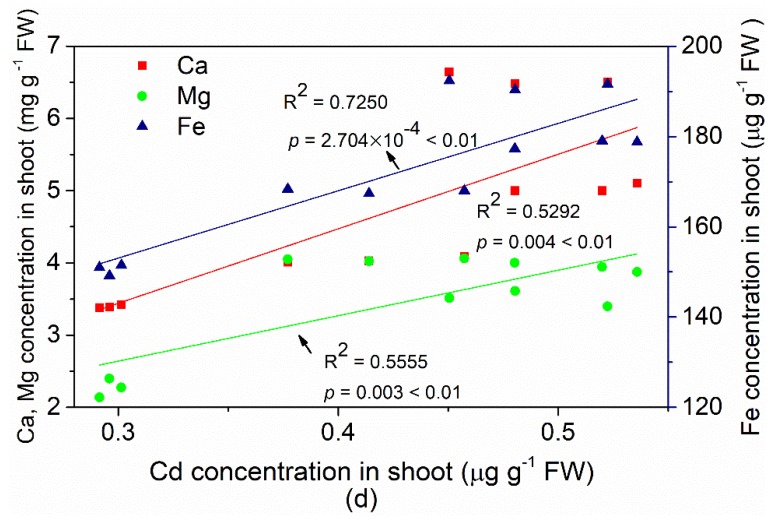
Ca (**a**), Mg (**b**), and Fe contents (**c**) (fresh weight) in the plants of control and exogenous glycinebetaine treatments and their relationships with Cd content (**d**) in the leaves after exogenous glycinebetaine treatment. Different lowercase letters indicate significant difference between treatments in leaf (ANOVA Duncan test, *p* < 0.05). Different capital letters indicate significant difference between treatments in root (ANOVA Duncan test, *p* < 0.05). The number of each group sample was five (*n* = 5).

Figure 4a–c show that after exogenous glycinebetaine treatment, the Ca, Mg and Fe contents in the leaves were significantly higher (*p* < 0.05) than those in the control, whereas Cd_leaf_, which denoted the Cd concentration in the leaves, was positively correlated with the Ca, Mg and Fe contents (*p* < 0.01) (Figure 4d). Cd is a non-essential element for plant growth that typically enters plants through Ca and Mg channels on the plasma membrane or zinc and ferric transporters [30,31,36,37]. For example, Cd can enter rice through Fe transporters [38] and the non-hyperaccumulating ecotype of *Sedum alfredii* through Ca channels [39]. These results implied that glycinebetaine increased the Ca, Mg, and Fe contents in the leaves; provided more shared absorption and translocation channels, such as Ca and Fe, for Cd; and enhanced the Cd uptake and accumulation in amaranth leaves.

### 3.4. Changes in the Pectin and Chlorophyll Contents and Their Relationship with the Cd Uptake and Accumulation in Amaranth Leaves

**Figure 5:**
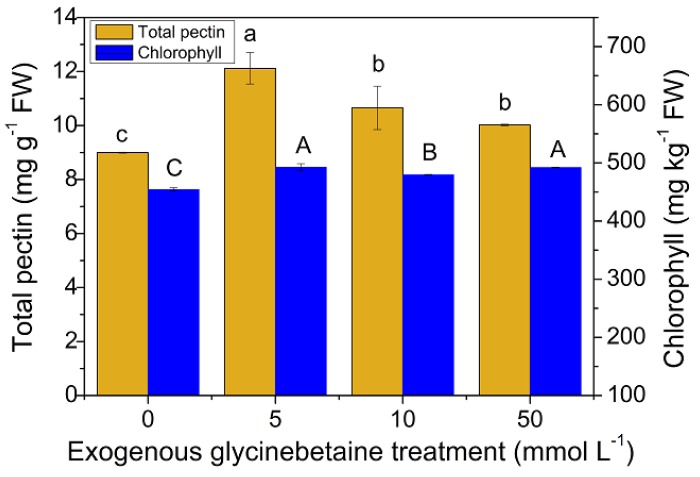
Pectin and chlorophyll contents (fresh weight) in leaves after exogenous glycinebetaine treatment. Different lowercase letters indicate significant difference between treatments for total pectin (ANOVA Duncan test, *p* < 0.05). Different capital letters indicate significant difference between treatments for chlorophyll (ANOVA Duncan test, *p* < 0.05). The number of each group sample was five (*n* = 5).

Figure 5 shows the pectin and chlorophyll contents in the leaves after exogenous glycinebetaine treatment. As shown in Figure 5, exogenous glycinebetaine treatment increased the chlorophyll content in the leaves. When the glycinebetaine concentration was 5 mM, the chlorophyll content increased by 8.4% relative to that in the control. This finding was consistent with the results reported in the literature, which stated that exogenous glycinebetaine treatment increases the chlorophyll content in tomatoes [40,41] and maize [6]. Glycinebetaine can improve the activity of various enzymes and photosynthetic pigments under high-temperature stress and can promote chlorophyll synthesis [9]. The leaf chlorophyll content was significantly positively correlated (*p* < 0.05) with the Mg and Fe contents (Table 3). Mg and Fe are important elements for chlorophyll enzymes. An increase in chlorophyll content increases the contents of chlorophyllase, Mg, and Fe. Therefore, we speculate that as glycinebetaine enhances photosynthesis and leaf chlorophyll synthesis, the demand for Mg and Fe in the leaves increases, thereby edible amaranth potentially induce the high expression of Mg and Fe absorption channel proteins. Mg and Fe share these channels with Cd, thereby promoting Cd accumulation in the leaves (Figure 4d).

Figure 5 also shows that exogenous glycinebetaine treatment significantly increased the pectin content in the leaves (*p* < 0.05). Pectin is a matrix polysaccharide in the cell wall, and it can selectively bind heavy metals to promote their accumulation in the leaves. Osmoregulatory substances can enhance the ability of pectin to bind heavy metals by altering polysaccharide metabolism and pectin composition [42,43]. An increase in the content of low methyl-esterified pectin in plant cell walls can increase the uptake and accumulation of heavy metals in plants [44]. Therefore, we speculate that exogenous glycinebetaine treatment increases the pectin content in the leaves, which further increases the Cd adsorption on the cell walls of leaves.

Table 3 shows that a significant positive correlation (*p* < 0.05) existed between the pectin and Ca contents in the leaves. Ca is the main body component of plant cell walls, and pectin calcium is formed in the intercellular layer of the cell walls. Therefore, increased pectin content increases the Ca content, which may induce high expression of Ca absorption channel proteins in the leaf cell membrane. Some of these channels have a low affinity and can also absorb Cd, promoting the Cd uptake in the leaves [30].

## 4. Conclusions

Exogenous glycinebetaine treatment on leaves in the hot season alleviated high-temperature stress but promoted the soil Cd uptake and accumulation by edible amaranth. Exogenous glycinebetaine treatment significantly increased the contents of soluble sugar, DOC, and LMWOAs in the rhizosphere soil solution (*p* < 0.05) and enhanced the soil Cd mobilization. In addition, exogenous glycinebetaine treatment significantly increased the chlorophyll and pectin contents in the leaves, which further increased the demand for Fe, Mg, and Ca. More Cd was absorbed and accumulated in the leaves through channels of these elements. These results indicated that exogenous glycinebetaine treatment in the subtropical hot season would increase the health risks of crops grown in Cd-contaminated soils.

## Figures and Tables

**Table 1:** Total Cd mobilization in the rhizosphere soils and the soluble sugar, dissolved organic carbon (DOC), and low-molecular-weight organic acids (LMWOAs) contents in the rhizosphere soil solution after exogenous glycinebetaine treatment.

GB/mM	Cd_total_ (µg pot^−1^)	Rhizosphere Soil Solution		
		DOC (µg g^−1^ DW)	LMWOAs (µg g^−1^ DW)	Soluble Sugar (µg kg^−1^ DW)
0	1.02 ± 0.02d	64.5 ± 1.01d	1.24 ± 0.14d	6.14 ± 0.21d
5	1.63 ± 0.01c	82.0 ± 1.23c	3.65 ± 0.11c	12.0 ± 0.10c
10	1.77 ± 0.04a	90.1 ± 0.80b	5.75 ± 0.29a	15.3 ± 0.45b
50	1.69 ± 0.02b	93.1 ± 0.20a	4.51 ± 0.21b	16.2 ± 0.19a
GB average	1.70	88.4	4.64	14.5
GB average/0 (fold)	1.66	1.37	3.73	2.36

GB: glycinebetaine. Different lowercase letters indicate significant difference in the column (ANOVA Duncan test, *p* < 0.05). The number of each group sample was five (*n* = 5). Values are presented as means ± SD.

**Table 2:** Correlations between total Cd mobilization (Cd_total_) in the rhizosphere soil and the soluble sugar, DOC, and LMWOAs contents in the rhizosphere solution.

Statistical Indicators	DOC	LMWOAs	Soluble Sugar
R^2^-value	0.874	0.867	0.855
*p*-value	0.043 < 0.05	0.045 < 0.05	0.049 < 0.05

The number of each group sample was five (*n* = 5).

**Table 3:** Correlations among pectin, chlorophyll, Ca, Mg, and Fe contents in the leaves after exogenous glycinebetaine treatment.

	Ca	Mg	Fe	Pectin	Chlorophyll
Ca	1				
Mg	0.095	1			
Fe	0.924 *	0.344	1		
Total pectin	0.856 *	0.171	0.448	1	
Chlorophyll	0.370	0.679 *	0.589 *	0.479	1

Asterisks (*) denote statistically significant correlations between the two factors (*p* < 0.05). The number of each group sample was five (*n* = 5).
